# Frequency-Specific Effects of Galvanic Vestibular Stimulation on Response-Time Performance in Parkinson's Disease

**DOI:** 10.3389/fneur.2021.758122

**Published:** 2021-11-02

**Authors:** Soojin Lee, Paul F. Smith, Won Hee Lee, Martin J. McKeown

**Affiliations:** ^1^Pacific Parkinson's Research Centre, University of British Columbia, Vancouver, BC, Canada; ^2^Wellcome Centre for Integrative Neuroimaging, Oxford Centre for Functional MRI of the Brain (FMRIB), Nuffield Department of Clinical Neurosciences, University of Oxford, Oxford, United Kingdom; ^3^Department of Pharmacology and Toxicology, School of Biomedical Sciences, University of Otago, Dunedin, New Zealand; ^4^Department of Software Convergence, Kyung Hee University, Yongin, South Korea; ^5^Faculty of Medicine (Neurology), University of British Columbia, Vancouver, BC, Canada

**Keywords:** Parkinson's disease, galvanic vestibular stimulation, stimulation frequency, response time, simple reaction time task

## Abstract

**Background:** Galvanic vestibular stimulation (GVS) is being increasingly explored as a non-invasive brain stimulation technique to treat symptoms in Parkinson's disease (PD). To date, behavioral GVS effects in PD have been explored with only two stimulus types, direct current and random noise (RN). The interaction between GVS effects and anti-parkinsonian medication is unknown. In the present study, we designed multisine (ms) stimuli and investigated the effects of ms and RN GVS on motor response time. In comparison to the RN stimulus, the ms stimuli contained sinusoidal components only at a set of desired frequencies and the phases were optimized to improve participants' comfort. We hypothesized GVS motor effects were a function of stimulation frequency, and specifically, that band-limited ms-GVS would result in better motor performance than conventionally used broadband RN-GVS.

**Materials and Methods:** Eighteen PD patients (PDMOFF/PDMON: off-/on-levodopa medication) and 20 healthy controls (HC) performed a simple reaction time task while receiving sub-threshold GVS. Each participant underwent nine stimulation conditions: *off-stimulation*, RN (4–200 Hz), ms-θ (4–8 Hz), ms-α (8–13 Hz), ms-β (13–30 Hz), ms-γ (30–50 Hz), ms-h1 (50–100 Hz), ms-h2 (100–150 Hz), and ms-h3 (150–200 Hz).

**Results:** The ms-γ resulted in shorter response time (RPT) in both PDMOFF and HC groups compared with the RN. In addition, the RPT of the PDMOFF group decreased during the ms-β while the RPT of the HC group decreased during the ms-α, ms-h1, ms-h2, and ms-h3. There was considerable inter-subject variability in the optimum stimulus type, although the frequency range tended to fall within 8–100 Hz. Levodopa medication significantly reduced the baseline RPT of the PD patients. In contrast to the off-medication state, GVS did not significantly change RPT of the PD patients in the on-medication state.

**Conclusions:** Using band-limited ms-GVS, we demonstrated that the GVS frequency for the best RPT varied considerably across participants and was >30 Hz for half of the PDMOFF patients. Moreover, dopaminergic medication was found to influence GVS effects in PD patients. Our results indicate the common “one-size-fits-all” RN approach is suboptimal for PD, and therefore personalized stimuli aiming to address this variability is warranted to improve GVS effects.

## Introduction

Parkinson's disease (PD) is a progressive disorder marked by the degeneration of dopaminergic neurons in the substantia nigra projecting to the basal ganglia (BG). As these neurons degenerate, individuals with PD frequently experience the cardinal motor symptoms of slowness of movement, tremor, rigidity, and postural instability. The estimated prevalence and incidence are expected to grow as a result of aging populations (1).

Dopamine-based pharmacologic treatments such as levodopa remain the gold standard for symptomatic treatment of PD (2) and are robust and effective in improving motor function, particularly in the early stages of the disease. However, some symptoms such as gait and balance dysfunction may be poorly responsive to dopaminergic medication (3), and many people who have been treated with levodopa for prolonged periods may experience complications such as dyskinesias and motor fluctuations (2). Deep brain stimulation (DBS) is an effective treatment for advanced PD (4) but utilized in as few as 2% of the PD population (5) for reasons including the invasiveness of surgical intervention and associated potential complications (6), medical comorbidities that prevent surgery, lack of advanced medical care, relatively mild symptoms, and good response to medication. The exact mechanisms underlying DBS effects are not yet fully understood, but likely involve suppression of pathological neural oscillations [e.g., exaggerated beta oscillations (7, 8)] in the BG circuit (9).

Inspired by the success of DBS in alleviating PD symptoms, non-invasive brain stimulation (NIBS) is being increasingly explored. As with DBS, NIBS techniques can apply electric currents to the brain to modulate neural activity (10, 11) and affect downstream behaviors (12). NIBS can be safely and economically tested within a wide range of the PD population, from early to advanced disease stages. Although NIBS lacks the ability to directly target focal areas for maximum effectiveness of the stimulation compared with DBS, it does not rely on implantable hardware. Hardware that must be implanted has severe constraints on design as it must be small in size, strongly conserve battery power, and have strict temperature regulation. In contrast, NIBS is not affected by these limitations to the same degree and can utilize external (and potentially portable) stimulators. Thus, NIBS techniques can employ more complicated stimulus waveforms such as random noise (RN) and multisine signals that can be delivered to achieve different effects, as we show here, as compared to electrical pulses used in DBS.

Galvanic vestibular stimulation (GVS) is one type of NIBS technique that applies weak electric currents to the mastoid processes behind the ears to modulate the firing rates of the vestibular afferents. In human studies, GVS has been utilized primarily as a means to activate the vestibular system in order to study balance and head movement responses (13). A pioneering study to investigate GVS effects on PD patients was conducted in 2005 (14) by applying 24-h continuous noisy GVS to six idiopathic PD patients and one patient with akinesia. The stimulation improved short-range heart rate variability, speed of transitions between rest and activity in the trunk, and reaction time in a Go/NoGo task.

Since then, GVS is being increasingly investigated for the treatment of PD symptoms, motivated by anatomical and functional evidence supporting close connections between the vestibular nuclei, thalamus, and BG (15–19). Prior GVS studies in PD have reported improvement in autonomic system regulation, postural balance and gait, and motor task performance (Supplementary Table 1). Notably, six out of the nine (66.7%) GVS studies have used RN stimuli while the other studies (33.3%) used direct current (DC) stimuli. The predominance of DC stimuli is likely because it has been long-used in balance research to induce body sways using GVS (20). Similarly, a RN stimulus has been adopted as it was used in the original GVS study in PD (14) and has been supported by the *stochastic resonance theory* stating that the addition of an appropriate level of random noise can paradoxically enhance the response of the nervous system to a weak signal (21–23). Notably, the GVS frequencies used in these studies have been limited to <30 Hz as this reflects the frequency range of most physical movements, and therefore likely reflects the physiological range of endogenous vestibular activation (24). However, we do not know if RN is the most effective stimulus and if different stimulus frequencies significantly influence the motor effects.

Here, we assessed the motor performance of PD participants in a simple reaction time task using several band-limited multisine GVS stimuli. Specifically, we compared whether the multisine stimuli can result in better task performance compared to the more traditional RN stimulus. We next sought to answer the following questions: (1) is there a single band-limited stimulus that brings about the most robust and largest effects across individuals?; (2) does the most effective band-limited stimulus vary across individuals?; and (3) how much improvement in motor performance can be evoked by varying stimulation frequency within an individual? Increasing evidence demonstrates that the same transcranial electrical stimulation can induce substantial variability in individual responses (25–28) due to various factors including methodological differences in the study protocols and participants' physiological traits (e.g., age and sex) and brain states (e.g., emotional and mental states) (28, 29). Here, we posit that a data-driven approach—whereby individual responses to different stimuli are assessed—may be a strategy to partly ameliorate these innate differences. Finally, for the first time, we aimed to address the question of whether GVS effects interact with levodopa medication by recruiting the same PD participants both off-/on-dopaminergic medication.

## Materials and Methods

### Participants

A total number of 20 PD patients and 22 age-matched healthy controls (HC) took part in this study. The study protocol was approved by the Clinical Research Ethics Board at the University of British Columbia. All participants gave written, informed consent prior to participation. No participant had any reported vestibular or auditory disorder, and all were right-handed. The PD patients were classified as having mild-stage PD (Hoehn and Yahr stage 1–2) without atypical Parkinsonism or other neurological disorders. Two PD participants and one HC participant did not complete the entire study protocol (see 2.2 Study protocol) due to extraneous reasons such as occasional coughing and arriving late for the experiment. As the motor task data collected from these subjects were ultimately incomplete, we excluded them from the data analysis. One HC participant was also excluded from the data analysis because the subject did not hold a pressure-sensor bulb as instructed and data were not usable when we subsequently inspected the data. Notably, no subjects failed to complete the entire study protocol due to the intolerability of the GVS.

In total, 18 PD and 20 HC participants were included in the data analysis (Table 1). The Unified Parkinson's Disease Rating Scale (UPDRS) Parts II and III were assessed for the PD patients in the off-medication state prior to the experiment.

**Table 1:** Demographic and clinical characteristics of the study participants.

	**PD (***n*** = 18)**	**HC (***n*** = 20)**
Age (years)	67.8 ± 7.3	68.7 ± 7.5
Gender (male/female)	9/9	10/10
Disease duration (years)	7.9 ± 4.4	–
UPDRS II	15.4 ± 8.2	–
UPDRS III	23.8 ± 9.7	–
- Bradykinesia^a^	9.3 ± 4.6	–
- Tremor^b^	8.0 ± 3.7	–
Hoehn and Yahr scale	1.3 (1–2)	–
Levodopa Equivalent Daily Dose (mg) (30)	731.3 ± 403.8	–

*Mean ± Standard deviation (SD)*.

a *Sum of the scores in UPDRS III 3.4–3.8 sections*.

b *Sum of the scores in UPDRS III 3.15–3.18 sections*.

### Study Protocol

In this present paper, we analyzed simple reaction time (SRT) task data collected as a part of a concurrent GVS-EEG study designed to investigate the effects of different GVS frequencies on: (1) cortical activity at rest; and (2) cortical activity and motor performance during the SRT task. In this section, we report the overall experimental procedure of the concurrent GVS-EEG study. The details of the SRT task are described in the next section.

**Figure 1:**
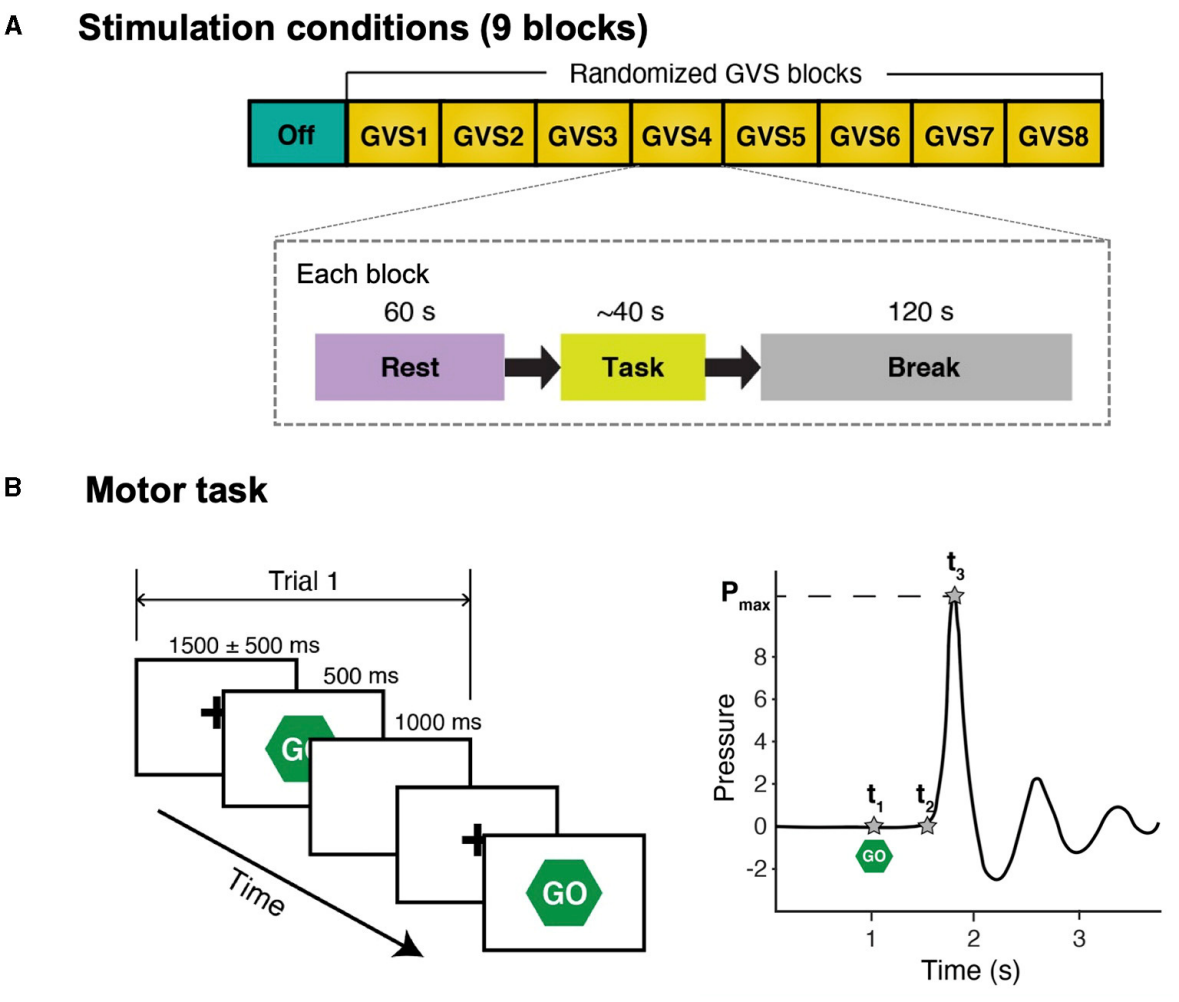
Schematic diagram of study protocol and simple reaction time task. **(A)** There were 9 stimulation conditions (blocks) and the order of GVS blocks was randomized for each participant. In each block, participants were instructed to stare at a fixation cross on a computer screen for 60 s (Rest) and perform a simple reaction time task afterward. There was a 120-s break between the blocks to avoid potential carry-over effects (i.e., GVS was not delivered during break). **(B)** Left: participants were instructed to respond to a visual cue (“Go”) as fast as possible by squeezing a pressure sensor bulb. There were 10 trials in each stimulation condition. For each trial, peak grip pressure (P_max_), time of visual cue (t_1_), time of movement onset (t_2_), and time of peak pressure (t_3_) were identified in the water pressure recording.

The experiment consisted of 9 blocks with different GVS conditions that were 2 min apart to minimize any confounding post-stimulation effects. Each block included a 60-s rest condition, followed by the SRT task (Figure 1A). Prior to the experiment, each participant's cutaneous threshold to GVS was measured (see section GVS). Then, the participants were fitted with an EEG cap. They were instructed to comfortably sit in front of a computer screen and focus their gaze on a continuously displayed fixed target for 60 s until they saw a written instruction to press a key on the keyboard to start the motor task. Further instruction on how to perform the SRT task was given, followed by a practice run consisting of 10 trials, and then the experiment began.

The PD participants performed the experiment in two sessions on the same day, in the off-medication (PDMOFF) and on-medication (PDMON) states. They stopped taking their normal levodopa medication and any dopamine agonists at least 12 and 18 h prior to the experiment, respectively. After the first session, they took their regular dose of medication and rested for an hour before beginning the second session. The HC participants performed the experiment once. At the end of the experiment, all the participants were verbally asked whether they felt any particular sensation or experienced pain, vertigo, nausea, or heat sensation at the stimulating electrodes in order to confirm the absence of placebo and adverse effects (13, 31).

### Simple Reaction Time Task

Participants were instructed to respond to a visual cue as fast as possible by squeezing a pressure-sensor bulb (Figure 1B). Each trial started with a hold phase in which a fixation cross was presented at the center for a randomized duration that ranged from 1,000 ms to 2,000 ms [N(1500, 500)]. Then, a visual cue (“Go”) appeared for 500 ms followed by a 1,000-ms white blank screen. The motor task in each stimulation block with the same stimulus consisted of 10 trials. A pressure-sensor bulb was used because it provides more descriptive behavior measures than a simple button-press and a prior study reported that PD patients demonstrate abnormal motor control while exerting pressure during a task of repeatedly squeezing a rubber bulb (32). The number of trials was selected such that the PD participants could still complete the entire study protocol without excessive tiredness (particularly during off-medication) while significant differences in task performance between conditions could still be detected.

The pressure was recorded at a sampling frequency of 250 Hz. For each trial, three temporal landmarks (*t*_1_, *t*_2_, and *t*_3_) were defined in the pressure recording (Figure 1B). Response time (RPT) was defined as *t*_3_−*t*_1_, which was divided into two subcomponents: (1) reaction time (RT) (*t*_2_−*t*_1_; the time between the stimulus onset and movement onset); and (2) movement time (MT) (*t*_3_−*t*_2_; the time required to execute the motor response), in order to further investigate whether GVS affects both RT and MT or only one of them exclusively. Mean RPT, RT, and MT across 10 trials in each block were computed for further statistical analyses.

### GVS

GVS was delivered in bilateral, bipolar fashion through pre-gelled Ag/AgCl electrodes (BIOPAC Systems Inc., USA) placed over the mastoid process behind each ear using a constant current stimulator DS5 (Digitimer, UK). We utilized systematic procedures previously used to determine individual cutaneous sensory threshold level (31, 33, 34). A noisy stimulus was delivered to the mastoid processes for 20 s at an imperceptible level, starting from a basal current level of 0.1 mA. The current intensity was then increased in 0.02 mA intervals until participants perceived a mild, local tingling sensation in the area of the stimulating electrodes for the duration of the stimulus. The current level was then decreased each time by one level until sensation was no longer reported and then increased by one step to confirm the threshold. In the experiment, GVS was applied at 90% of the determined threshold value to avoid the effects of placebo, general arousal, and/or voluntary selective attention.

In each stimulation block (Figure 1A), either random noise (RN; 4–200 Hz) or a band-limited multisine stimulus was delivered. A multisine stimulus was adopted as it has the advantages of reducing experiment time by testing multiple sinusoids at once and preserves the power spectrum over a frequency range of interest without any spectral leakage compared with random noise (35). A multisine signal *x*(*t*) can be expressed as:


$$\begin{array}{l} x\left(t\right)=A\overset{N_{k}}{\underset{k=1}{\sum }}\sin\left(\omega_{k}t+\phi_{k}\right) \end{array}$$

where *A* is the amplitude, *N*_*k*_ is the number of sinusoidal components, ω_*k*_ and ϕ_*k*_ are the frequency and phase of each sinusoidal component *k*, respectively.

**Figure 2:**
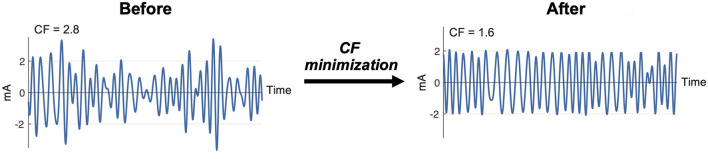
Clipping algorithm to minimize crest factor (CF) of a multisine signal.

Seven multisine stimuli were designed in total. ms-θ, ms-α, ms-β, and ms-γ were designed to cover conventional EEG frequency bands, and ms-h1, ms-h2, and ms-h3 to cover high frequency ranges (Table 2). For each stimulus, the sinusoidal frequencies (ω_*k*_) were uniformly distributed every 0.4 Hz (e.g., the ms-β consisted of sinusoids at 13.0, 13.4, …, 29.8 Hz). The phases (ϕ_*k*_) of the sinusoids were chosen to minimize the crest factor using a clipping algorithm (36) (Figure 2) to increase signal-to-noise ratios and improve participants' comfort (37, 38).

**Table 2:** Frequency ranges of the GVS stimuli investigated in the study.

	**RN**	**ms-θ**	**ms-α**	**ms-β**	**ms-γ**	**ms-h1**	**ms-h2**	**ms-h3**
Frequency (Hz)	4–200	4–8	8–13	13–30	30–50	50–100	100–150	150–200

The active-GVS blocks were randomly ordered for each participant, and the *off-stimulation* block was conducted before any active-GVS block to enable a comparison of motor performance between the PD and HC groups without any carry-over stimulation effects. In this study, behavioral effects of GVS were investigated using only the active-GVS blocks that were completely randomized.

### Statistical Procedures

GVS thresholds between groups were compared using the two-sample *t*-test, and correlations between GVS thresholds and age or clinical scores were tested using the Pearson correlation coefficient.

For each group, the RPTs during active-GVS blocks were compared using a one-way repeated measures analysis of variance (RM-ANOVA) with STIM (RN, ms-θ, ms-α, ms-β, ms-γ, ms-h1, ms-h2, and ms-h3) as a within-subject factor. To investigate the interaction effect of GVS and medication for PD participants, we additionally conducted an overall two-way RM-ANOVA with STIM and MED (on and off) as within-subject factors. Mauchly's test was used to assess the ANOVA assumption of sphericity, and the Greenhouse-Geisser correction was used if necessary to correct for non-sphericity. When a significant effect was found, *post-hoc* pairwise comparisons with the Bonferroni correction were conducted. If there was a stimulus that evoked a significantly different RPT compared with RN-GVS, the RT and MT were compared using a paired *t*-test.

All statistical analyses were performed using IBM SPSS (version 27). Significance was assigned to *P* < 0.05.

## Results

None of the participants reported any adverse effects nor awareness of any differences between stimulation conditions.

### GVS Threshold

There was no significant difference between the PD and HC groups in GVS threshold level [PD: 0.50 ± 0.24 mA; HC: 0.46 ± 0.18 mA; *t*_(36)_ = 0.56, *P* = 0.58]. The thresholds of all participants were not significantly correlated with age (*r* = 0.06, *P* = 0.73), but there was a significant sex difference [males = 0.51 ± 0.21 mA; females = 0.35 ± 0.13 mA; *t*_(36)_ = 2.89, *P* = 0.006]. For PD participants, no significant correlations were found between their thresholds and clinical scores (disease duration: *r* = −0.11, *P* = 0.69; UPDRS III: *r* = 0.22, *P* = 0.39; bradykinesia: *r* = 0.35, *P* = 0.16; tremor: *r* = −0.19, *P* = 0.44).

### Effects of GVS Frequencies on RPT

**Figure 3:**
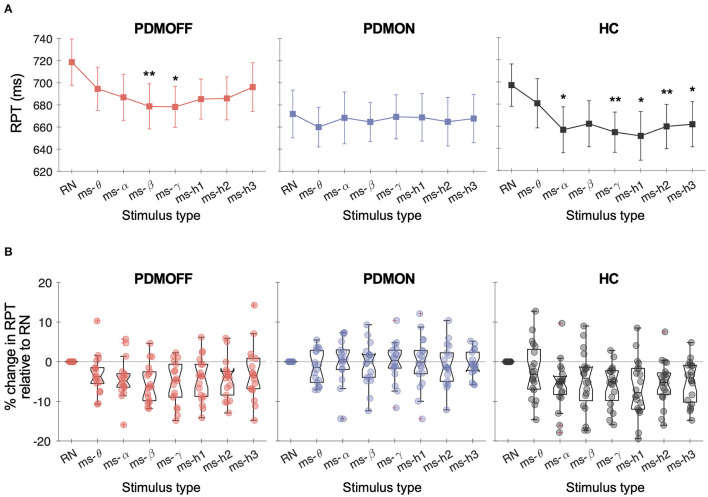
**(A)** Response time (RPT) measured in different GVS conditions. Markers and vertical lines represent the group mean and standard error of the mean (SEM), respectively. *P* values from the *post-hoc* tests are indicated (*P < 0.05 and **P < 0.01 compared to RN). **(B)** % change in the RPT compared to RN-GVS. Each dot represents a participant.

A one-way RM-ANOVA revealed a main effect of STIM for the PDMOFF [*F*_(7, 119)_ = 4.38, *P* < 0.001] and HC [*F*_(7, 133)_ = 5.97, *P* < 0.001] groups, but not for the PDMON group [*F*_(7, 119)_ = 0.356, *P* = 0.93] (Figure 3A). For the PDMOFF group, *post-hoc* tests found that RPT was significantly shorter during ms-β (*P* = 0.008) and ms-γ (*P* = 0.026) compared to RPT during RN, and the % change of RPT was −5.5 ± 4.8 and −5.4 ± 5.4%, respectively (Figure 3B). No significant change in RPT was observed for ms-θ (*P* = 0.51), ms-α (*P* = 0.11), ms-h1 (*P* = 0.093), ms-h2 (*P* = 0.056), and ms-h3 (*P* = 1.0). For the HC group, *post-hoc* tests revealed that, compared to RN, the RPT significantly decreased during ms-α (*P* = 0.01), ms-γ (*P* = 0.001), ms-h1 (*P* = 0.012), ms-h2 (*P* = 0.008), and ms-h3 (*P* = 0.013), but not during ms-θ (*P* = 1.0), and ms-β (*P* = 0.24) (Figure 3A).

We further investigated the nature of the significant RPT changes (Table 3). For the PDMOFF group, ms-β significantly decreased RT (*P* = 0.0095) but not MT (*P* = 0.17) whereas ms-γ decreased MT (*P* = 0.013) but not RT (*P* = 0.076). For the HC group, RT was decreased by ms-h1 (*P* = 0.013) and ms-h2 (*P* = 0.048). Overall, the results did not indicate that variation of GVS frequency evokes an exclusive change in RT or MT.

**Table 3:** *Post-hoc* comparisons of RT and MT measured during GVS.

	**PDMOFF**		**HC**	
**Stimulus**	**RT (ms)**	**MT (ms)**	**RT (ms)**	**MT (ms)**
RN	478.0 ± 16.1	240.8 ± 15.7	472.3 ± 9.9	225.0 ± 17.8
ms-θ	N/A	N/A	N/A	N/A
ms-α	N/A	N/A	452.9 ± 10.0 (*P* = 0.09)	204.0 ± 18.0 (*P* = 0.069)
ms-β	450.6 ± 13.2 (*P* = 0.0095)	228.1 ± 15.3 (*P* = 0.17)	N/A	N/A
ms-γ	456.6 ± 13.9 (*P* = 0.076)	221.6 ± 14.1 (*P* = 0.013)	449.1 ± 10.6 (*P* = 0.065)	205.9 ± 16.8 (*P* = 0.097)
ms-h1	N/A	N/A	445.7 ± 10.0 (*P* = 0.013)	205.7 ± 18.2 (*P* = 0.065)
ms-h2	N/A	N/A	450.4 ± 8.6 (*P* = 0.048)	209.3 ± 18.2 (*P* = 0.20)
ms-h3	N/A	N/A	454.0 ± 11.3 (*P* = 0.14)	207.8 ± 18.2 (*P* = 0.15)

*Values are shown only for the RN and multisine stimuli that resulted in significant RPT change compared to RN*.

*P values from paired t-tests (multisine vs. RN) are shown. N/A, Not applicable*.

The two-way RM-ANOVA revealed a main effect of STIM [*F*_(7, 119)_ = 2.78, *P* = 0.01] and MED [*F*_(1, 17)_ = 5.59, *P* = 0.03]. Although PD participants tended to benefit from multisine stimuli more during off-medication state (Figure 3B), the STIM × MED interaction effect did not reach the statistical significance [*F*_(7, 119)_ = 2.03, *P* = 0.056].

### Effects of Time Order on RPT

To assess whether there was any spurious time-order effect on RPT, we re-arranged RPTs in chronological order for every participant and performed a one-way RM-ANOVA with TIME as a within-subject factor.

**Figure 4:**
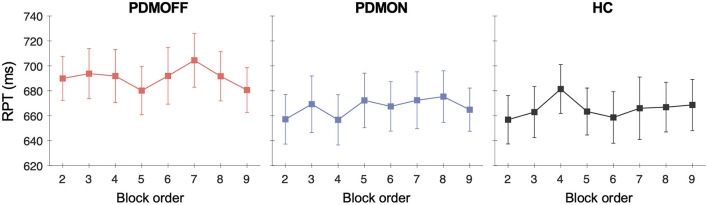
Group mean and SEM of the RPTs that are temporally ordered as opposed to stimulus types. The x-axis represents the temporal order of the stimulation blocks. The first *off-stimulation* block (Figure 1A) is not shown.

No effect of TIME was found for all three groups [PDMOFF: *F*_(7, 119)_ = 1.31, *P* = 0.253; PDMON: *F*_(3.8, 64.8)_ = 1.38, *P* = 0.251; HC: *F*_(7, 133)_ = 1.14, *P* = 0.341] (Figure 4).

### Sensitivity of RPT on GVS Frequency

We investigated whether the degree of RPT variation induced by different stimuli differed between the PDMOFF, PDMON, and HC groups. To quantify this, for each participant, we computed the standard deviation (RPT_SD_) and range (RPT_range_; max–min) of RPTs across the eight stimulation blocks.

Group comparisons suggested that both RPT_SD_ and RPT_range_ were comparable between the PDMOFF and HC groups (Table 4). PDMON participants showed relatively smaller RPT_SD_ and RPT_range_ compared to the other two groups, but these differences did not reach statistical significance.

**Table 4:** Comparisons of RPT variability (RPT_SD_ and RPT_range_) across the eight GVS conditions.

	**Mean ± SD**			**P**		
	**PDMOFF**	**PDMON**	**HC**	**PDMOFF − PDMON**	**PDMOFF − HC**	**PDMON − HC**
RPT_SD_ (ms)	27.2 ± 9.9	23.2 ± 10.7	29.8 ± 11.2	0.15	0.45	0.074
RPT_range_ (ms)	82.2 ± 29.9	68.8 ± 31.4	89.6 ± 33.3	0.084	0.48	0.057

*P values obtained from student t-tests are presented*.

### Intersubject Variability in Most and Least Effective Stimuli

**Figure 5:**
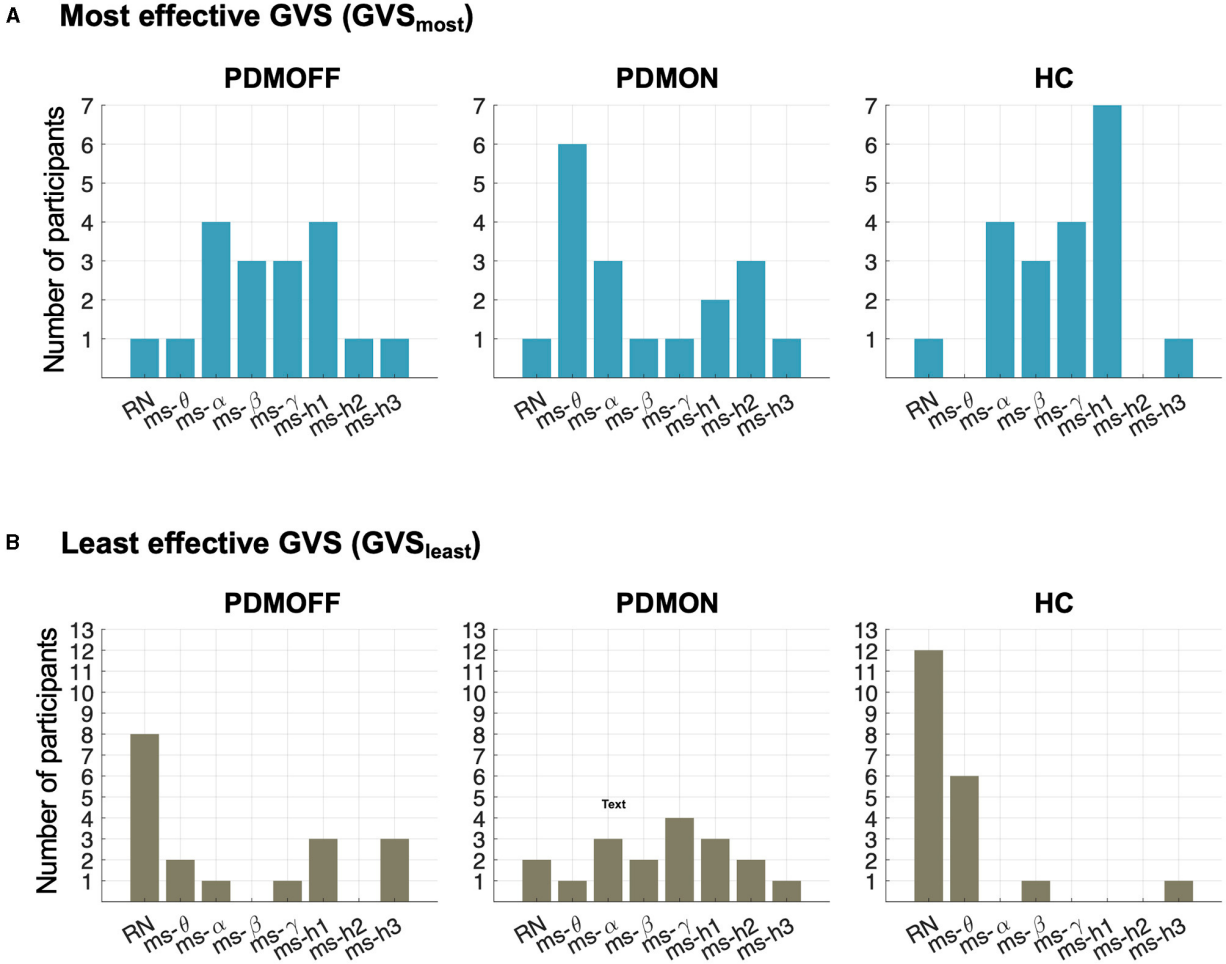
Distributions of stimulus types during which RPT was the shortest (GVS_most_; top) and the longest (GVS_least_; bottom) for the participants in each group.

Figure 5A shows the distributions of the most effective stimulus (GVS_most_) that resulted in the shortest RPT for each participant. Interestingly, the distributions of the PDMOFF and HC groups appeared similar in that 77.8 and 90.0% of the participants, respectively, showed their best task performance during ms-α, ms-β, ms-γ, or ms-h1. By comparison, only 38.9% of the PDMON participants performed the best in these frequency ranges.

The contrast between the PDMON and the other two groups was also observed when we investigated the least effective stimulus (GVS_least_) that resulted in the longest RPT (Figure 5B). RN and ms-θ were found to be the least effective stimuli for 55.5 and 90.0% of the PDMOFF and HC participants, respectively. On the other hand, only 16.7% of the PDMON participants showed their worst performance during RN and ms-θ.

### Significance of the RPT Decrease by GVS_most_

**Figure 6:**
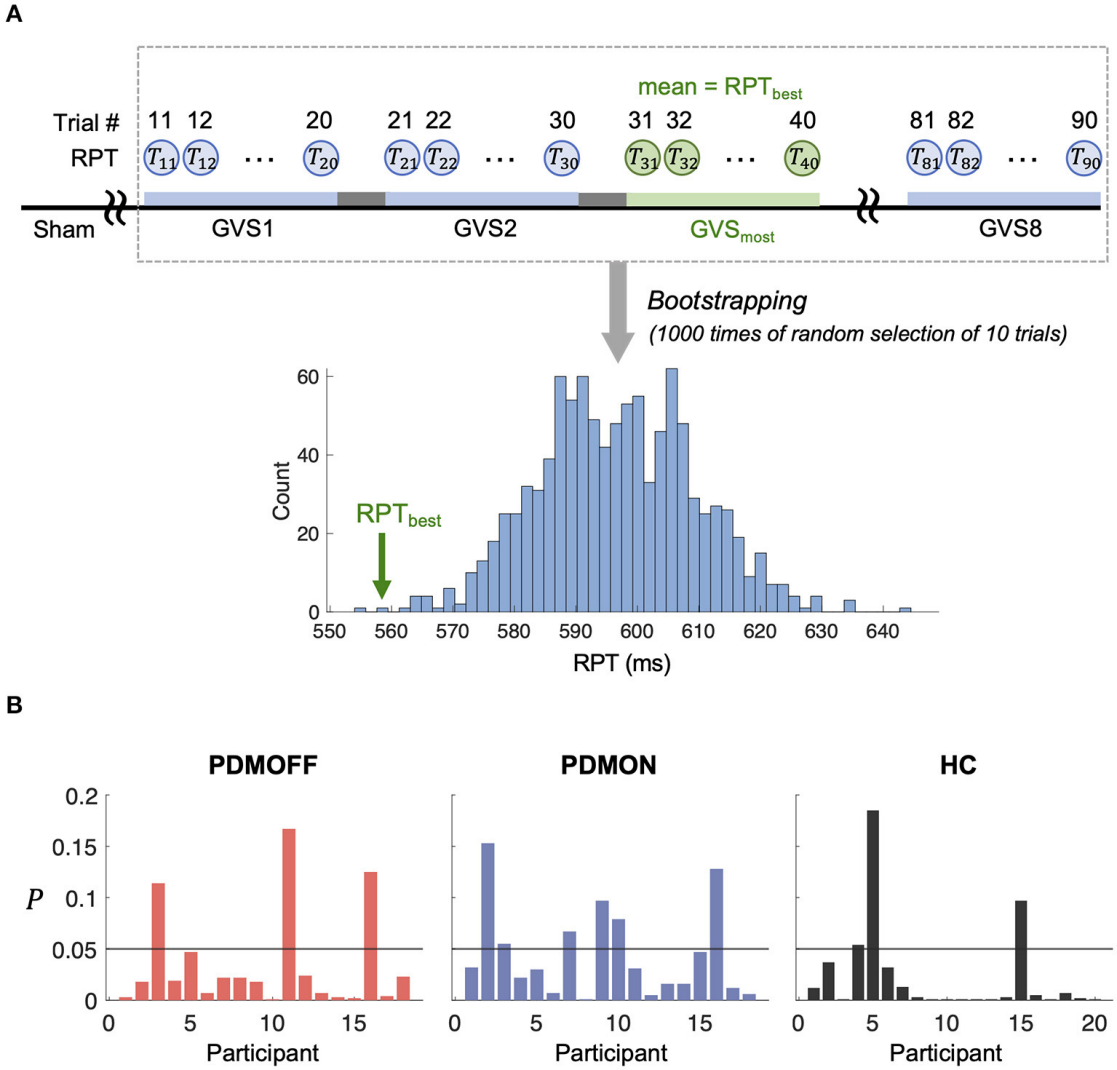
**(A)** Schematic diagram showing a process to generate an empirical distribution of RPT for each participant. Each bootstrap sample randomly selects RPTs of 10 trials and the mean of the bootstrap samples is computed to generate the distribution. RPT_best_ represents the mean RPT during GVS_most_. **(B)** *P* value of RPT_best_ computed based on **(A)** is shown for every participant.

To assess whether the RPT evoked by GVS_most_ was significantly faster compared with the other stimuli, we computed its *P* value based on the empirical distribution of RPT estimated by a bootstrapping approach (Figure 6A). Note that as the RPT is computed as the mean over 10 randomly selected trials, it can still be shorter than the mean RPT during GVS_most_. Figure 6B shows that 83.3, 66.7, and 85% of the PDMOFF, PDMON, and HC participants, respectively, exhibited significantly shorter RPT during GVS_most_ (*P* < 0.05) compared with the expected RPT during any GVS stimulus.

## Discussion

To our knowledge, this is the first study investigating SRT task performance of PD and HC participants while applying GVS across a wide range of frequencies. Overall, our results suggest that RPT can be improved by GVS in PD patients. However, as bradykinesia is a key feature in PD, we cannot disentangle whether or not the RPT improvements were a result of faster decision-making and/or faster movement. This will require examining the simultaneously acquired EEG and will be the topic of another report. We demonstrated that the motor improvement is significantly dependent on the GVS parameters used. Surprisingly, we found that RN-GVS, despite its popularity, did not actually evoke the best task performance in the PDMOFF and HC groups, with ms-γ (30–50 Hz) proving superior in reducing RPT (Table 5). The performance of the off-medicated PD participants during ms-β and ms-γ were comparable to the baseline performance when they were in the on-medication state. We found that the GVS frequency that resulted in the shortest RPT varied considerably across participants, suggesting that a one-size-fits-all stimulus will not be as effective as a personalized stimulus. For most of the PDMOFF and HC participants, the best GVS frequency varied in the range of 8–100 Hz. The worst task performance was found during RN or ms-θ for more than half of the participants in these two groups. These results provide evidence that further work is required to tailor GVS parameters for maximum efficacy.

**Table 5:** Summary of RPTs (unit: millisecond) measured during *off-stimulation* (baseline), RN-GVS, and multisine GVS for the PD and HC participants.

	**PD (***n*** = 18)**	**HC (***n*** = 20)**
**Off-medication**		
Baseline	748.5 ± 93.8	674.4 ± 107.5
GVS	RN: 718.7 ± 88.5 ms-β: 678.7 ± 86.6** ms-γ: 678.2 ± 78.3*	RN: 697.2 ± 85.4 ms-α: 656.8 ± 92.7* ms-γ: 654.6 ± 81.2** ms-h1: 651.3 ± 98.2* ms-h2: 659.8 ± 89.5** ms-h3: 661.8 ± 91.0*
**On-medication**		
Baseline	683.2 ± 92.4	N/A
GVS	RN: 671.82 ± 91.6	N/A

*For multisine GVS, only those that resulted in significant changes compared with RN-GVS are displayed (*

* *P < 0.05 and*

** *P < 0.01 compared with RN as in Figure 3). N/A, Not applicable*.

Whether or not RPT is actually delayed in PD has been controversial (39–41), partially due to methodological heterogeneity and different clinical characteristics of the participants (42). In this study, the difference in baseline RPT between the PDMOFF and HC groups did not reach statistical significance (*P* = 0.068; Supplementary Table 2). Instead, the most interesting finding was that responses to different GVS stimuli showed a similar trend between the two groups (Figure 3). This finding may suggest that there are some mechanisms underlying the GVS effects that are common between these groups.

In contrast, we found that the PDMON group showed relatively different responses to GVS. Normally, dopamine is active both phasically and tonically during motor performance. Levodopa has complex effects in PD, which may result in relative normalization of tonic dopamine firing, yet impairment of phasic firing (43). While phasic dopamine firing is normally associated with rewards in reinforcement learning paradigms, it may also be involved in internal representations of desired actions with actual sensory feedback during motor performance (44–46). Thus, many studies have suggested that movement-related phasic changes can be observed in nigrostriatal dopamine neuron firing (47–49), and that dorsal striatal phasic dopamine signaling is associated with specific kinematic features of movement (43). Complex effects of dopaminergic medication in PD have also been reported in fMRI studies (50–54) showing that levodopa medication does not simply restore brain connectivity aberrant in PD. Rather it induces functional connectivity changes distinctive from those identified to be different between PD patients and healthy controls (51, 54). Taken together, this is an important point to consider for future GVS studies, as prior studies (Supplementary Table 1) included only medicated PD patients, and the information on the dosage and timing of the medication was rarely reported.

Given the functional role of pathological beta oscillations in PD (55), the result of particular interest was that ms-β resulted in the largest decrease in RT among the tested stimuli in the PDMOFF group whereas it did not improve motor performance for the HC group. In this regard, there is some evidence to support the concept that beta-frequency stimulation may have clinical effectiveness in PD patients. In a transcranial alternating current stimulation (tACS) study conducted on 10 PD and 10 HC participants (56), 20-Hz stimulation at the primary motor cortex (M1) yielded a significant decrease in beta-band cortico-muscular coupling in PD patients but not in HC. A TMS study showed that 20–33 Hz stimulation at M1 elicited significant suppression of the motor evoked potential (MEP) in PD patients and the amount of suppression was correlated with their UPDRS III scores (57). It should be noted, however, that there have been only a handful of studies that utilized beta-frequency NIBS in PD patients, and it is difficult to determine from our results whether the ms-β effects observed in the PD participants were related to the pathological beta-band activity. Thus, further neuroimaging studies are strongly suggested to be carried out to validate our results and elucidate the mechanisms of action.

The frequency-dependent GVS effects we observed may be related to the overlap between the neural processes affecting RPT and neural pathways affected by external vestibular inputs. One of the main vestibular pathways is the direct ascending projection from the vestibular complex to the thalamus, primarily targeting the ventral anterior, ventral lateral, ventral posterior lateral, ventral posterior medial, intralaminar nuclei, and the geniculate bodies (58–60). Strong activations in these regions by vestibular stimulation (18, 59, 61–63) suggest a critical thalamic contribution to processing vestibular information (60, 63). The ventral parts of the thalamus are also closely connected with M1, premotor cortex, and BG (15, 58, 63, 64), modulating a range of aspects in motor control (15, 63, 65). Thus, we conjecture that GVS effects on RPT can be in part explained by vestibular inputs affecting the motor thalamus. This may also explain the mild GVS effects on the PDMON participants as the BG inputs to the motor thalamus would vary at different dopamine levels.

It is also possible that GVS affected the striatum, a region described as an integrative center for sensory information and involved in motor planning and execution. Although the largest inputs to the striatum are from the cortex, recent studies have elucidated the subcortical pathways critical for interpreting and responding to environmental stimuli (66, 67). Electrophysiological studies in animal models and neuroimaging studies in humans have shown that vestibular stimulation activates the head of the caudate nucleus and putamen (16–19, 62, 68, 69), likely through the parafascicular thalamic nucleus (PFN) (64, 70). In addition, it has been recently proposed that the striatal tail may play a role as a multisensory integration center (71), and thus it is possible that there are vestibular inputs to this region as well.

Our observations of different motor effects evoked by varying GVS frequency are consistent with many animal studies (72, 73). Surprisingly, canal and otolith afferents in macaque monkeys responded to GVS as a function of frequency such that the response gain (i.e., spikes/s/mA) increased more than twice when the stimulation frequency varied from 0.1 to 25 Hz (72). This seminal finding opposes the common idea that high-frequency GVS would result in *smaller* gains because the tissues between the electrode and vestibular afferents may act as a low-pass filter. Similarly, the firing rate of the PFN increases when the frequency of stimulation applied to the semicircular canal nerve is >100 Hz (73). Taken together, these findings could explain in part the efficacy of the frequency range we observed in most of the PDMOFF and HC participants.

There are several limitations in our study. Considering the study design and our primary objective to examine different types of GVS stimuli, we did not try to replicate previous findings demonstrating that GVS results in better motor performance compared to baseline performance seen during *off-stimulation*. Although we think the practice effect on the task performance is unlikely for a simple, over-learned motor task like ours, the possibility was not completely ruled out when *the baseline measurement* always preceded active-GVS. Similarly, our study was not designed to measure the after-effects of GVS. Post-stimulation behavior effects of GVS are largely unknown (13). Studies that examined GVS aftereffects stimulated participants for more than 30 min, and the results are conflicting (74–77). The issue of whether stimulation effects last after the cease of stimulation is not only limited to GVS but is one of the main controversial topics for transcranial electrical stimulation (78). As online stimulation effects differ depending on stimulation parameters (e.g., frequency, intensity, duration, target sites) and experimental tasks, the presence and duration of after-effects appear to be influenced by the stimulation parameters and tasks (79, 80). Although after-effects are infrequently reported, evidence from tES studies shows the presence of after-effects when stimulation was applied at >0.5 mA for longer than 10 min (78, 81–83). Given that we applied GVS for a short duration at a low current intensity with a 2-min inter-block off-stimulation break, we suggest that any effects carried over from previous stimulation were relatively mild compared to the online-stimulation effects. Validation of GVS after-effects and their relationships with stimulation parameters will be areas/topics of interest for future work. Note that, since GVS can utilize portable stimulators, reliance on much more subtle after-effects is not as important as other technologies that are not as easily portable (e.g., TMS). Finally, several studies support the notion that GVS effects are mostly spatially restricted to the vestibular organs. For instance, the auditory effects of GVS are rare (20) despite the proximity between the auditory and vestibular systems. GVS evokes circumscribed cortical activation of vestibular areas, and effects on the somatosensory cortex are only seen at specific frequencies (62). At higher intensities, the stimulation of the vestibular system can be self-reported by feelings of vertigo. A recent computational modeling study of the electric field generated by GVS (84) suggests that the bilateral and bipolar configuration, as used in our study, results in the most spatially-restricted current flow to the vestibular organs. However, some current may diffuse to the medulla, pons, and cerebellum. Although we note that both the electrodes (11 mm) and current intensity used here (0.43 ± 0.19 mA) were less than those used in the computational model (30 mm and 1 mA), we cannot completely discount that some of our results may be via modulation of extra-vestibular structures.

In conclusion, our findings provide key information necessary for the future development of GVS techniques to induce robust and effective therapeutic effects in PD. Future research is warranted to confirm similar behavioral effects of GVS applied at frequencies beyond the assumed physiological ranges and to establish potential mechanisms.

## Data Availability Statement

The raw data supporting the conclusions of this article will be made available by the authors, without undue reservation.

## Ethics Statement

The studies involving human participants were reviewed and approved by Clinical Research Ethics Board at the University of British Columbia. The patients/participants provided their written informed consent to participate in this study.

## Author Contributions

SL designed and conducted the study, including patient recruitment, data collection, and data analysis. MJM participated in the study design, data analysis, supervision, and funding acquisition. All authors have participated in manuscript writing and editing and approved the final manuscript.

## Funding

SL was supported by Rina M. Bidin Foundation Fellowship in Research of Brain Treatment. WL was supported by the National Research Foundation of Korea (NRF) grant funded by the Korea government (MSIT) (No. 2021R1C1C1009436). MJM was supported by John Nichol Chair in Parkinson's Research and the Canadian Institutes of Health Research (CIHR) grant (453374).

## Conflict of Interest

The authors declare that the research was conducted in the absence of any commercial or financial relationships that could be construed as a potential conflict of interest.

## Publisher's Note

All claims expressed in this article are solely those of the authors and do not necessarily represent those of their affiliated organizations, or those of the publisher, the editors and the reviewers. Any product that may be evaluated in this article, or claim that may be made by its manufacturer, is not guaranteed or endorsed by the publisher.
