# Supplementary material for: Frequency-Specific Effects of Galvanic Vestibular Stimulation on Response-Time Performance in Parkinson's Disease
Source: Front Neurol. 2021 Nov 2;12:758122. doi: 10.3389/fneur.2021.758122 (PMC8593161; doi:10.3389/fneur.2021.758122)
Supplement: Supplementary file 1 [file Data_Sheet_1.DOCX]

Supplementary Material

**Supplementary Table S1.** Summary of GVS studies reporting behavioural effects in PD (adapted from (1)).

| **Year** | **Stimulus** | **Current intensity** | **Duration** | **Participants** | **Main finding** |
| --- | --- | --- | --- | --- | --- |
| 2005  (2) | 1/f noise  (0.01–2 Hz) | Subthreshold | 24 hr | 6 PD, 1 PA,  8 MSA | - Improved autonomic system regulation - Decreased reaction time to visual cue |
| 2008  (3) | 1/f noise  (0.01–2 Hz) | Subthreshold | 24 hr | 3 PD, 1 PA,  8 MSA, 2 CCA | - Increased frequency and speed of switching between higher and lower levels of wrist activity |
| 2009  (4) | 1/f noise  (0.01–2 Hz) | 0.1, 0.3, 0.5 mA | 26 s | 5 PD, 20 HC | - Decreased body sway with eyes closed |
| 2015  (5) | White noise  (< 30.0 Hz) | Subthreshold | $\leq$ 3 hr | 10 PD | - Improved balance corrections - Reduction in postural response time |
| 2015  (6) | 1/f noise  (0.1–10 Hz) | Subthreshold | During task | 12 PD | - Reduced overshooting and noisy movements in a visuomotor tracking task |
| 2015  (7) | DC | 0.2–0.7 mA | 20 min | 7 PD | - Improved bending posture |
| 2016  (8) | DC | 0.7 mA | 20 min | 5 PD | - Improved postural stability |
| 2018  (9) | DC | 2 $\times$ threshold | During task | 11 PD | - Improved variation of the step duration in gait - Improved finger tapping task performance |
| 2018  (10) | White noise  (0.4–30 Hz) | Subthreshold | 60 s | 13 PD, 12 HC | - Changes in posture and increased sway amplitude - Mildly decreased sway frequency |

Abbreviations: PD = Parkinson’s disease, PA = pure akinesia, MSA = multiple system atrophy, CCA = cortical cerebellar atrophy, HC = healthy control

**Supplementary Table S2**. Comparisons of RPT during sham-GVS between groups. $P$ values obtained from student $t$-tests are presented.

|  | **Mean** $\boldsymbol{\pm}$ **SD** | | |  | $\boldsymbol{P}$ | | |
| --- | --- | --- | --- | --- | --- | --- | --- |
|  | **PDMOFF** | **PDMON** | **HC** |  | **PDMOFF**  $\boldsymbol{-}$ **PDMON** | **PDMOFF**  $\boldsymbol{-}$ **HC** | **PDMON**  $\boldsymbol{-}$ **HC** |
| RPT (ms) | 748.5 $\pm$ 93.8 | 683.2 $\pm$ 92.4 | 674.4 $\pm$ 107.5 |  | 0.0044 | 0.068 | 0.82 |

Supplemental Citations

1. Lee S, Liu A, McKeown MJ. Current perspectives on galvanic vestibular stimulation in the treatment of Parkinson’s disease. *Expert Rev Neurother* (2021) **21**:405–418. doi:10.1080/14737175.2021.1894928

2. Yamamoto Y, Struzik ZR, Soma R, Ohashi K, Kwak S. Noisy vestibular stimulation improves autonomic and motor responsiveness in central neurodegenerative disorders. *Ann Neurol* (2005) **58**:175–181. doi:10.1002/ana.20574

3. Pan W, Soma R, Kwak S, Yamamoto Y. Improvement of motor functions by noisy vestibular stimulation in central neurodegenerative disorders. *J Neurol* (2008) **255**:1657–61. doi:10.1007/s00415-008-0950-3

4. Pal S, Rosengren SM, Colebatch JG. Stochastic galvanic vestibular stimulation produces a small reduction in sway in Parkinson’s disease. *J Vestib Res* (2009) **19**:137–42. doi:10.3233/VES-2009-0360

5. Samoudi G, Jivegård M, Mulavara AP, Bergquist F. Effects of stochastic vestibular galvanic stimulation and LDOPA on balance and motor symptoms in patients with Parkinson’s disease. *Brain Stimul* (2015) **8**:474–480. doi:10.1016/j.brs.2014.11.019

6. Lee S, Kim DJ, Svenkeson D, Parras G, Oishi MMK, McKeown MJ. Multifaceted effects of noisy galvanic vestibular stimulation on manual tracking behavior in Parkinson’s disease. *Front Syst Neurosci* (2015) **9**:5. doi:10.3389/fnsys.2015.00005

7. Okada Y, Kita Y, Nakamura J, Kataoka H, Kiriyama T, Ueno S, Hiyamizu M, Morioka S, Shomoto K. Galvanic vestibular stimulation may improve anterior bending posture in Parkinson’s disease. *Neuroreport* (2015) **26**:405–10. doi:10.1097/WNR.0000000000000360

8. Kataoka H, Okada Y, Kiriyama T, Kita Y, Nakamura J, Morioka S, Shomoto K, Ueno S. Can postural instability respond to galvanic vestibular stimulation in patients with Parkinson’s disease? *J Mov Disord* (2016) **9**:40–43. doi:10.14802/jmd.15030

9. Khoshnam M, Häner DMC, Kuatsjah E, Zhang X, Menon C. Effects of galvanic vestibular stimulation on upper and lower extremities motor symptoms in Parkinson’s disease. *Front Neurosci* (2018) **12**:633. doi:10.3389/fnins.2018.00633

10. Tran S, Shafiee M, Jones CB, Garg S, Lee S, Pasman EP, Carpenter MG, McKeown MJ. Subthreshold stochastic vestibular stimulation induces complex multi-planar effects during standing in Parkinson’s disease. *Brain Stimul* (2018) **11**:1180–1182. doi:10.1016/j.brs.2018.04.020
